# The Importance of Non-accessible Crosslinks and Solvent Accessible Surface Distance in Modeling Proteins with Restraints From Crosslinking Mass Spectrometry*[Fn FN2]

**DOI:** 10.1074/mcp.M116.058560

**Published:** 2016-05-05

**Authors:** Joshua Matthew Allen Bullock, Jannik Schwab, Konstantinos Thalassinos, Maya Topf

**Affiliations:** From the ‡Institute of Structural and Molecular Biology, Birkbeck College, University of London, Malet street, London, WC1E 7HX, UK;; §Gene Center Munich, Ludwig-Maximilians-Universität (LMU) Munich, Feodor-Lynen-Strasse 25, 81377 Munich, Germany;; ¶Institute of Structural and Molecular Biology, Division of Biosciences, University College London, London WC1E 6BT, UK

## Abstract

Crosslinking mass spectrometry (XL-MS) is becoming an increasingly popular technique for modeling protein monomers and complexes. The distance restraints garnered from these experiments can be used alone or as part of an integrative modeling approach, incorporating data from many sources. However, modeling practices are varied and the difference in their usefulness is not clear. Here, we develop a new scoring procedure for models based on crosslink data—Matched and Nonaccessible Crosslink score (MNXL). We compare its performance with that of other commonly-used scoring functions (*Number of Violations* and *Sum of Violation Distances*) on a benchmark of 14 protein domains, each with 300 corresponding models (at various levels of quality) and associated, previously published, experimental crosslinks (XLdb). The distances between crosslinked lysines are calculated either as Euclidean distances or Solvent Accessible Surface Distances (SASD) using a newly-developed method (Jwalk). MNXL takes into account whether a crosslink is nonaccessible, *i.e.* an experimentally observed crosslink has no corresponding SASD in a model due to buried lysines. This metric alone is shown to have a significant impact on modeling performance and is a concept that is not considered at present if only Euclidean distances are used. Additionally, a comparison between modeling with SASD or Euclidean distance shows that SASD is superior, even when factoring out the effect of the nonaccessible crosslinks. Our benchmarking also shows that MNXL outperforms the other tested scoring functions in terms of precision and correlation to Cα-RMSD from the crystal structure. We finally test the MNXL at different levels of crosslink recovery (*i.e.* the percentage of crosslinks experimentally observed out of all theoretical ones) and set a target recovery of ∼20% after which the performance plateaus.

Protein structure determination is key to the mechanistic understanding of proteins and by extension, the cell. However, determining the structure of a given protein is often very challenging. Traditional techniques like x-ray crystallography, NMR spectroscopy or cryo-electron microscopy, which can lead to high-resolution structures, cannot always be used or require a large amount of experimental effort (1–3). Protein structures can instead be modeled computationally, incorporating a variety of experimental information from different sources to arrive at a consensus model (4). This process can start by creating a comparative model of the target protein if the structure of a homologous protein (template) is available (5–6). Even with sequence identity as low as 15% the structure of the protein is often conserved (7) and comparative modeling can be used.

The difficulty however, is in measuring the accuracy of the resulting models. In order to identify accurate models, experimental data can be used. Such an approach has been shown to be useful, for example, in constraining comparative models with electron microscopy density maps, leading to the first model of a mammalian ribosome (6, 8). One increasingly popular source of constraining information comes from chemical crosslinking mass spectrometry (XL-MS) (9, 10). The method has some major advantages compared with conventional methods. It is much less sensitive to protein contamination, only a small amount of protein is needed (11) and even *in vivo* crosslinking can be performed (12). Although it only provides low-resolution restraints, recent applications have given deep insights into the structure of various proteins and protein complexes (5, 9, 12–15).

The basic concept behind chemical crosslinking is simple and well established. A crosslinker consists of two reactive groups joined together by a carbon linker of a specified length. The reactive groups can be designed to target a variety of amino acids including those with amino or carboxyl side-chains or they can be less specific. The most commonly used crosslinkers, bissulfosuccinimidyl suberate (BS3) and disuccinimidyl suberate (DSS) (11), target amino groups, *i.e.* lysine residues and the N terminus. The soluble protein is reacted with a crosslinker, which covalently links two residues that fall within the linker-length distance from each other (16). After enzymatic digestion the linked peptides can be detected by MS. Due to the low abundance of crosslinked peptides compared with noncrosslinked peptides, the detection of crosslinks is not straightforward. The percentage of experimentally observed crosslinked peptides, compared with the total theoretically possible can be low. We refer to this percentage as the recovery rate and previous studies suggest that this rate varies between 8% to 20% (17). However, a variety of methods, *e.g.* enrichment by means on SCX or SEC peptide fractionation, are being developed to increase such recovery rates (18).

Crucially, in order to react with the linker reagents, the side chains also need to be surface accessible and the linker must traverse the surface of the protein to reach the other side chain. Simply calculating the Euclidean distance between two lysine residues does not provide an accurate description of the distance over which a crosslink will form as most of the time other parts of the protein will obstruct this path (19, 20).

Previous studies have used the solvent accessible surface distance (SASD)^1^ instead, as implemented in the *Xwalk* software (http://www.xwalk.org/). The SASD is defined as the shortest possible path between two amino acids without penetrating the protein's surface. Another approach of calculating the distance between crosslinked residues is to make an approximation of SASD (21). Here the protein surface is substituted by a sphere and the SASD approximated by the arced distance between the two crosslinked residues.

Despite currently being the most popular tool for calculating SASDs, Xwalk has some major drawbacks; the first being speed. The average time taken to run a 10 kDa protein with 10 lysines is 40 s using a 3.1 GHz Intel Core i7 MacBook Pro. While this is not a problem for validating crosslinks gathered experimentally, it is prohibitively slow when being used for large scale modeling purposes that generate tens to hundreds of thousands of models. The second drawback is that, during the running of experiments detailed herein, Xwalk was observed to generate solvent accessible paths that travel through the protein mass. This reduction in the confidence one is able to put in Xwalk's output compelled us to write our own SASD calculation algorithm, based on the same theoretical principles, called Jwalk (see Materials/Methods) which parallelizes the problem to achieve significant speed increases.

The maximum SASD between two amino acids about which a crosslink can form has not yet been unambiguously defined. As it is more robust to measure inter-residue distances from the backbone rather than the side chain, the crosslink will have a maximum physical length resulting from the linker length plus the length of the side chains. For example, DSS, which has a length of 11.4 Å, will have a maximal physical length of 22.4 Å, given the length of a lysine side chain is ∼5.5 Å (11.4 + 2*5.5). However, much longer distances between crosslinked residues are commonly observed (17) and recent publications use a maximum-bound of 35 Å as a restraint when modeling (5, 11, 22). This discrepancy between theoretical and observed lengths has been explained by the structural flexibility of the protein, but molecular dynamics simulations challenge the extent of this flexibility, suggesting a maximum-bound of 30 Å (23). As of yet there is no full explanation or consensus within the field.

The XLdb, a database of experimentally observed crosslinks, contains information curated from several crosslinking experiments in the literature. From this database the distribution of the distances between crosslinked residues, both Euclidean distances and SASD can be visualized as a density distribution (10, 17). This experimental distribution therefore provides additional information that can be exploited when developing a scoring method. All distances in the database were calculated with Xwalk. We also provide our own recalculated version of the database using Jwalk.

Most examples in the literature use crosslinking restraints by totaling up the number of times the distance between crosslinked residues exceeds the maximum-bound (*Number of Violations*) (5, 14, 22, 24). Variations on this scoring method employ a smoothing function around the maximum bound to avoid having a distinct cut-off distance (21, 25). Summing the excess distance for each pair of crosslinked residues if the distance between them exceeds the maximum-bound (*Sum of Violation Distances*) has also been used as a complementary approach (9). There is however no consensus in the literature to which distance type should be used. Even though using the Euclidean distance is theoretically flawed, it is significantly easier to compute than the SASD. To our knowledge, no direct comparison to judge the effect of using one distance over the other has been performed.

In order to better model proteins using crosslinks, we have developed a new scoring method to evaluate models, which takes advantage of the experimentally observed distribution of distances between crosslinked residues in XLdb, and used it to investigate the effects of using SASD over Euclidean distance. We begin by giving an overview of our SASD calculation algorithm, *Jwalk* (Methods). We then describe our scoring function, matched and non-accessible crosslink score (MNXL), that scores each crosslink gathered from crosslinking experiments based on its calculated SASD. The scoring procedure takes into consideration the probability that a crosslink of a given length will appear in a protein structure, while also penalizing crosslinked residues that exceed the maximum-bound or are not solvent exposed. We then compare the performance of this scoring function with previously used approaches by applying it to a benchmark of proteins (from XLdb), each containing multiple models of varying quality generated by comparative modeling (Results). The implications of these results are then discussed as well as why some proteins appear to be more amenable to crosslink guided modeling than others (Discussion).

## 

### 

#### Experimental Procedures

#### Jwalk

In order to accurately calculate the SASD between two residues, the Jwalk algorithm was written. The approach our algorithm takes is similar to that of Xwalk, although our parallelized implementation is more efficient (see Results). Jwalk is standalone software and can be downloaded either as part of TEMPy software (26) or from http://topf-group.ismb.lon.ac.uk/Jwalk/Jwalk.tar.gz.

The calculation of the SASD between two residues by Jwalk can be divided into three main phases (Fig. 1): (1) placement of the protein(s) onto a grid; (2) calculation of the solvent accessible surface; and (3) a Breadth-First Search of the grid to calculate the shortest possible SASD between the all residues of interest.

**Fig. 1.**
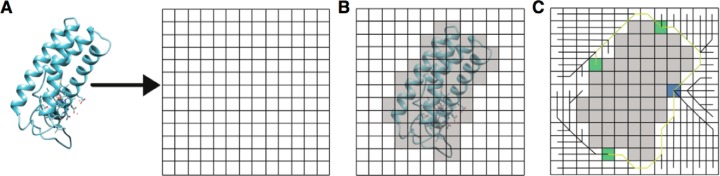
**Overview of the three main phases of the Jwalk algorithm.** *A*, placement of the protein(s) onto a grid; *B*, calculation of the solvent accessible surface by expanding a sphere around each atom; and *C*, a Breadth-First Search of the grid to calculate the shortest possible SASD between the all residues of interest. The search is initiated from one of the surface lysines (blue square) with potential paths (black lines) searching the whole grid. Paths between the starting lysine and targets lysines (green squares) are retained and output as a .pdb file (yellow paths). Fig. 1. (location: Experimental Procedures - Jwalk).

First, the atomic coordinates of the protein are parsed and a surrounding grid of zeroes is generated by taking the maximum and minimum values in each xyz plane to create an array just large enough to contain the protein - 4 voxels each way (Fig. 1*A*). Each atomic coordinate is then mapped to the nearest grid point. The default voxel size is 1 Å.

The solvent accessible surface (SAS) is then approximated by expanding a sphere around each atom in the structure except those of the lysine and N-terminal side-chains (Fig. 1*B*). The radius of the sphere is the VDW radius of the atom in question plus half the VDW radius of a water molecule (1.4/2 = 0.7 Å) rounded to the nearest voxel size (to avoid missing borderline exposed residues that would be marked as buried due to the blurring that occurs at 1 Å voxel resolution). All grid points within these spheres are given a value of 1 and no inter-residue path is able to travel through this space. Solvent accessible lysines and N termini are then identified by expanding a similar sphere around the Nζ (and Cα atoms in case the sidechain is missing) of each lysine side-chain (Cα only for N termini). If any voxels within the sphere have a value of zero, then the residue is defined as solvent accessible and the voxel is added to a list of potential starting voxels for that lysine with regards to the later search.

A Breadth-First Search algorithm is then performed between solvent accessible lysines as well as between the N-terminal residue and lysines (Fig. 1*C*). The whole grid is iteratively searched, adding adjacent voxels to the queue each time, with the iterations ending once the whole grid has been searched or a maximum distance has been reached (in this study we set no maximum limit to SASD calculation in order calculate all possible SASDs). This search is performed starting from each surface accessible lysine and the N terminus. An additional connection is made between the surface accessible voxel and the Cα containing voxel. The shortest paths between each are then extracted from the full grid search.

Jwalk outputs two files: (1) A list of all inter-lysine/N-terminal SASDs; and (2) a .pdb file containing the shortest paths between each residue pair Cα. The code has been implemented to allow for parallelization if a multi-core processor is used. This results in significant speed gains over Xwalk (an approximate 1:1 ratio of proportional decrease in calculation time against number of processors used *e.g.* 36.37 s in Jwalk *versus* 119.5 s in Xwalk for benchmark example 1BLF_5–333_ with 25 lysines running on a 3.1 GHz Intel Core i7 quad core).

#### 2.2 Matched and Non-accessible Crosslink Score

Given a protein model in an ensemble of models, the SASD between all possible lysine pairs (Cα to Cα) are calculated to create the test data set. These are then compared with the experimental MS data set of residue pairs that have experimentally observed crosslinks or a theoretical MS data set of residue pairs that have an SASD of less than 33 Å. The cut-off of 33 Å was determined empirically (see Results). Matched and Nonaccessible Crosslink Score (MNXL) scores crosslinks by placing them in one of two categories, matched crosslink or nonaccessible crosslink, with scores depending on the distances between crosslinked residues (Eq. 1). The categories are taken from the four possible outcomes of the comparison between SASDs of the modeled data (test data set) and the experimental data (MS data set), described below. In each model, the scores (described below) corresponding to each crosslinked residue pair are totaled into a final score.

#### Matched Crosslink

If the SASD between a pair of lysines in the MS data set can be calculated in the model, then it is defined as a matched crosslink and scored as follows:

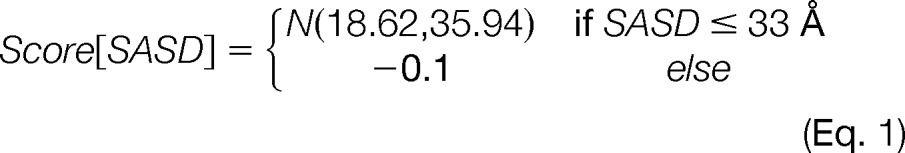
 If the SASD is under 33 Å, it is scored positively, taking into account its probability distribution, which is given by a normal distribution (the mean and variance are calculated from all the SASDs ≤33 Å from the XLdb). If the SASD exceeds 33 Å (indicating inconsistency with the native structure) it is scored with a flat penalty of −0.1.

#### Nonaccessible Crosslink

If the SASD between a pair of lysines in the MS *data set* cannot be calculated, then it is defined as a nonaccessible crosslink. This will happen if there is a loss of surface accessibility caused by minor conformational changes, resulting in a “blocked” path. Alternatively, this could also be the result of the SASD exceeding the maximum distance calculated by Jwalk, however in this study no maximum distance was used. This category therefore captures information separate to the distance information typically used from crosslinking data. To penalize for a nonaccessible crosslink a flat penalty of −0.1 is used.

#### Other Possible Outcomes

There are two more possible outcomes when comparing the data set with the model. One of these considers pairs of lysines that have a calculated SASD but are not present in the experimental data (MS data set). It can be risky to penalize such events because MS experiments only detect the most abundant crosslinks, with a typical coverage of below 20% (9, 10, 17). Thus, a crosslink between these two residues might actually be viable in the native structure. Therefore, we decided not to use this possibility.

The final outcome is if a crosslink is present in neither the model nor the data set that could possibly exist in the crosslinked protein. This outcome has no informational value.

For the purposes of this study, the total score is normalized between 0 and 1, with 0 given to the best score in the ensemble, in order to make direct comparisons between the different scores easier.

#### Other Crosslink Scoring Methods

Previously in the literature, two different crosslink scoring approaches have been used, number of violations (NoV) (24) and sum of violation distances (SoVD) (9). We applied both of these approaches in order to compare them to our newly developed scoring function.

#### Number of Violations

Crosslinks are scored with either 0 or 1 depending on whether the distance between crosslinked residues is below or above the cut-off, respectively. In this study, we used an SASD cut-off of 33 Å. The distances between each crosslinked residue pair in the model are scored and totaled into one final score, with a lower score pertaining to a more near-native model.

#### Sum of Violation Distances

Here, crosslinks are scored by taking the violating distance over 33 Å for each violating crosslink. These are then totaled for the model into the sum of violation distances, also with a lower sum pertaining to a more near-native model.

#### Experimental Design and Statistical Rationale

Our scoring function MNXL groups crosslinks into either *matched* or nonaccessible crosslinks, which are then scored based on their SASDs or given a flat penalty, respectively. We then compare MNXL to two other scoring functions seen in the literature, NoV and SoVD, using an experimental benchmark taken from XLdb.

#### Comparative Model Test Benchmark

In order to evaluate our scoring function, we tested it on a benchmark of 10 proteins, dividing some cases into individual domains/subunits to give 14 single domains in total. All the proteins are from XLdb with the criterion for selection being only monomers or dimers with a minimum of five crosslinks (per subunit). For each protein, 300 models were generated in MODELLER (27). The models were purposefully generated at different levels of accuracy using the 'suboptimal alignment' function combined with the 'automodel function' to span a range of 0–30 Å Cα-RMSD (28). Single chain proteins that were made up of two domains attached by a flexible loop (1BLF and 2D3I) were divided into two and each domain was modeled separately (interdomain crosslinks were removed from the data set). Four cases in the benchmark are protein dimers; in these cases, each subunit was modeled separately and no attempt was made to model the final complex (intersubunit crosslinks were removed from the data set).

#### Testing Crosslink Coverage Dependence

We wanted to evaluate what influence the crosslink coverage has on scoring function performance. Therefore, we tested all three scoring functions on each benchmark protein using MS data sets of 90/80/70/60/50/40/30/20/10/5 and 1% of all possible crosslinks. To ensure that we obtained a representative sample of possible MS data sets, 1000 random data sets were bootstrapped for each percentage value. We scored all the models against all data sets. This was done for all three scoring methods (MNXL, NoV, and SoVD).

#### Score Assessment

For a scoring function to be effective, there needs to be a good correlation between the score and how native-like the models are. To assess the latter, Cα-RMSD was used. The Pearson product-moment correlation coefficient was used to assess the correlation. An “ideal” scoring function should have the following characteristics: a correlation coefficient of 1.0 between Cα-RMSD and the score toward the correct fit, the native structure scoring best, and the score of the best model being significantly separate (+2σ) from the mean score of the ensemble (29).

In addition to the Cα-RMSD, the precision of each scoring function was calculated. We define Precision as TP/(TP+FP). A true positive (TP) is defined as a model that is ranked in the top-20 and has an RMSD ≤ 4 Å. A false positive (FP) is defined as a model that is ranked in the top-20 but has an RMSD value of > 4 Å. These were then averaged across the benchmark. If many models cluster on the same score they will all be ranked the same, which makes selecting the top-20 and calculating the precision problematic. Therefore, 20 random samples were taken from the cluster and the precision calculated; this was repeated 1000 times to generate an average result.

#### Results

#### Recalculating SASDs from XLdb

XLdb is a database of experimentally observed crosslinks that has been curated from the literature (10). The original published database contains information on 571 crosslinks across 57 atomic structures. This includes the residue and chain IDs of the crosslinked residues, whether the crosslink is intra- or inter- subunit, the Euclidean distance, the SASD and the crosslinker used (all crosslinkers used were 11.4 Å in length). Distributions of both SASD and Euclidean distances between experimentally observed crosslinked residues therefore provide information that can be used to predict the probability of observing a given inter-residue distance within a model and is the basis for the positive scoring aspect of our crosslink scoring function.

Given that we found Xwalk to calculate some erroneous SASDs, we ran Jwalk on all the proteins in XLdb. This revealed that many entries in the database, which contained residues that neither existed nor were present in the crystal structure, were crosslinked to themselves, or were not lysines or N terminus, or were listed as duplicates. In all of these cases, Euclidean distances and SASDs were still listed. These entries were subsequently removed to create a Jwalk-curated XLdb. Additionally, nearly all of the distances in the XLdb (Euclidean and SASD) were calculated from Lys-Cβ to Lys-Cβ, however, most crosslink cut-off distances used in the literature refer to Cα-Cα distances (10, 13, 17, 24).

The two distributions (XLdb and Jwalk-curated XLdb) are statistically different (*p* value 2.251 × 10^−5^ and supplemental Fig. S1), with the Jwalk distribution being slightly shifted to the right (*i.e.* longer SASDs). This is mainly because of the fact that the distances are now exclusively between Cα-Cα, with no Cβ-Cβ. There is also a notable difference at <10 Å where in the original XLdb there are two separate populations, which merge into one distribution in the Jwalk-curated XLdb. This population is attributed to crosslinks between neighboring lysines on alpha helices. Inspection of these SASDs shows that many are calculated incorrectly by Xwalk with the path traveling through the protein instead of across its surface (supplemental Fig. S2). The Jwalk-curated XLdb can be downloaded from http://topf-group.ismb.lon.ac.uk/Jwalk/XLdb_curated.xlsx.

#### Filtering Comparative Models Using MNXL

In order to evaluate our scoring function—matched and non-accessible crosslink score (MNXL)—and compare it to the other scoring methods available, we ran it on a benchmark of 14 single protein domains/subunits taken from XLdb, each with corresponding 300 comparative models (Table I), using their experimentally observed crosslinks. MNXL was initially tested against two other scoring methods: number of violations (NoV) and sum of violation distances (SoVD) (see Methods).

**Table I:** Location: Results - Filtering comparative models using MNXL

PDB	Chain (Residue Range)	No. of Lysines	Correlation			Precision		
			MNXL	NoV	SoVD	MNXL	NoV	SoVD
1BLF	A (5–333)	25	0.65	0.03	0.11	0.75	0.07	0.05
1BLF	A (334–689)	28	0.62	0.22	0.02	0.55	0.05	0.05
1HRC	A (1–104)	19	0.42	0.22	0.36	0.50	0.10	0.15
1MBO	A (1–153)	19	0.39	−0.03	0.01	0.20	0.06	0.06
1JM7	A (1–103)	10	0.25	0.01	0.08	0.40	0.17	0.17
1U6R	A (1–380)	34	0.57	0.33	0.21	0.25	0.19	0.19
1U6R	B (1–380)	35	0.51	0.07	−0.26	0.20	0.00	0.00
1UJZ	B (447–573)	18	0.44	0.17	0.19	0.60	0.16	0.16
2D3I	A (5–335)	28	0.65	−0.08	−0.01	0.50	0.00	0.00
2D3I	A (342–686)	30	0.74	0.03	−0.05	0.70	0.03	0.05
2HGD	A (3–230)	18	0.79	0.70	0.63	0.35	0.07	0.07
4FGF	A (20–143)	12	0.27	0.14	0.25	0.10	0.00	0.00
4F5S	A (1–583)	59	0.80	0.53	0.51	0.35	0.25	0.00
4F5S	B (1–583)	59	0.55	0.01	−0.07	0.10	0.10	0.00
		AVERAGE	0.55	0.17	0.14	0.40	0.09	0.07
		S.D.	0.17	0.21	0.23	0.20	0.07	0.07

Despite a few difficult cases, MNXL performs reasonably well, with an average correlation against Cα-RMSD of 0.55 and average precision (*i.e.* the proportion of models ranked in the top-20 with an Cα-RMSD < 4 Å) of 0.40. Overall, MNXL is the best performing score both in terms of correlation with Cα-RMSD and precision systematically performing better on every test-case over the benchmark (Table I).

Fig. 2 shows for three cases in the benchmark (PDB ids: 1BLF_5–333_, 1JM7:A, and 4FGF) the Cα-RMSD of each model to the corresponding experimentally determined structure versus the crosslink score, for the three different crosslink scores (MNXL, NoV, and SoVD). 1BLF_5–333_ is the best performing protein in the benchmark because of having 25 surface lysines and 10 crosslinks distributed across the whole protein, which provides a comprehensive set of restraints to successfully model with. 1JM7:A is a protein that performs badly primarily down to having only four experimentally observed crosslinks (recovery of 16.12%) all concentrated within one region of the protein. Therefore, large deviations from the experimentally-determined structure are tolerated as no crosslinks are violated. (Fig. 2*B*: blue versus. beige structures - both satisfy all the experimental crosslinks). 4FGF is the worst performing protein in the benchmark, despite having 17 experimentally observed crosslinks across 12 surface lysines. The reasons for this are not obvious but it is likely to be a result of several factors; (1) 7 of the 17 recovered crosslinks exceed the SASD maximum-bound of 33 Å—suggesting native flexibility; (2) 10 of the crosslinks involve either Lys^110^, Lys^119^ or Lys^125^, all of which are in the same long loop region; (Fig. 2*B*: loop highlighted in red) and (3) the crosslinks only cover one half of the protein.

**Fig. 2.**
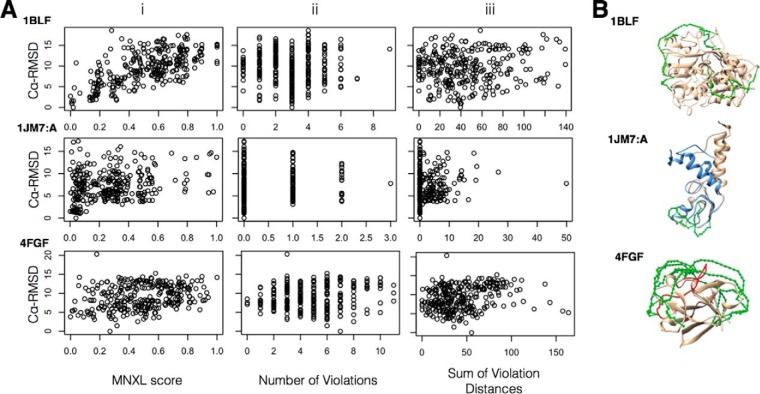
***A*, Plots showing the correlation between (i) MNXL, (ii) NoV and (iii) SoVD, and Cα-RMSD of three of the members of the benchmark with PDBid: 1BLF_5–333_, 1JM7:A and 4FGF and corresponding comparative models.** The ideal trend would stretch from the bottom left corner to the top right (see corresponding correlations values in Table I); *B*, The experimentally determined structures with SASDs mapped on (green) as well as additional highlighting (1JM7:A - blue structure highlights the structural deviations that are possible while still satisfying the crosslinking restraints; 4FGF - The majority of the crosslinks come from the red loop spanning residues 110–125). Fig. 2. (location: Results - Filtering homology models using MNXL).

By breaking down MNXL into its constituent components, the increase in performance over NoV and SoVD was revealed to be primarily down to the non-accessible crosslink aspect (see Methods). Scoring the benchmark by totaling just the non-accessible crosslinks gives a correlation of 0.39 and precision of 0.21 (Fig. 3). When this total is added to the number of violating crosslinks and used to score the benchmark, the correlation becomes very similar to MNXL (0.55 versus 0.53). However, the complete MNXL score is still better in terms of precision (0.40 versus 0.35).

**Fig. 3.**
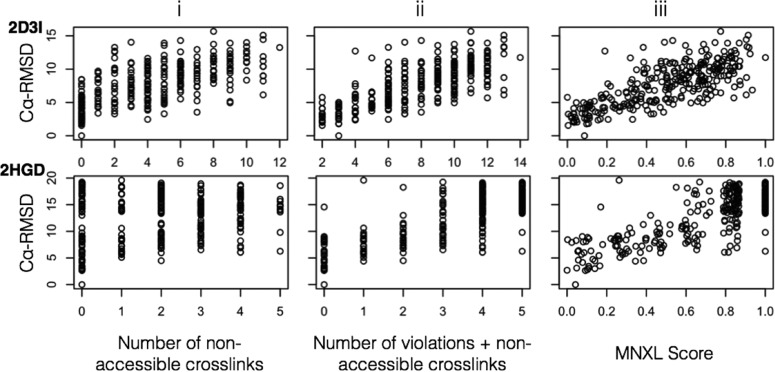
**The constituent elements of MNXL.** Benchmark members with PDBid: 2D3I_342–686_ and 2HGD scored with (i) non-accessible crosslinks only, (ii) SASD NoV and non-accessible crosslinks, and (iii) MNXL. Fig. 3. (location: Results - Filtering comparative models using MNXL).

#### Euclidean Distance versus SASD

If only the Euclidean distance between crosslinked lysines is calculated, there is no concept of non-accessible crosslinks, as no consideration is made as to whether the lysine is surface accessible or not. Although, to our knowledge, not previously seen in the literature, it is possible to calculate the Euclidean distances only between surface accessible lysines and therefore retain this information.

In order to specifically investigate the effect of using SASD against Euclidean distance we compared both types of “NoV with non-accessible crosslinks” scores (while Euclidean distances were *only* calculated between surface lysines) (Fig. 4i-ii). The use of Euclidean distance over SASD results in a drop in correlation from 0.55 to 0.42 and drop in precision from 0.35 to 0.17 (over the entire benchmark), respectively. This is reflected in the plots that show increased bunching of high Cα-RMSD models around the lowest (*i.e.* best) scores. If the Euclidean distance is used without considering surface accessibility or nonaccessible crosslinks, then the correlation and precision drop dramatically to 0.08 and 0.06, respectively (Fig. 4iii).

**Fig. 4.**
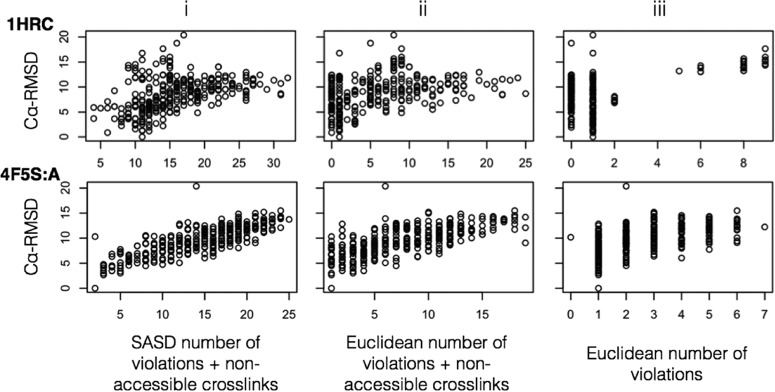
**The effect of modeling using Euclidean distances instead of SASD, highlighted with plots from two members of the benchmark - PDBid: 1HRC and 4F5S:A - scored with (i) SASD NoV and non-accessible crosslinks, (ii) Euclidean NoV and non-accessible crosslinks, and (iii) Euclidean NoV only.** Fig. 4. (location: Results - Euclidean Distance *versus* SASD).

#### Exploring Crosslink Coverage

Given that the average experimental recovery of crosslinks (based on XLdb) is ∼22%, we wanted to observe how dependent the performance of MNXL is on crosslink recovery. We tested all of the scoring methods on bootstrapped data sets of 90/80/70/60/50/40/30/20/10/5 and 1% of a full theoretical data set. The average correlation and precision at each percentage recovery across the benchmark can be seen in Fig. 5. The trend is nonlinear as the percentage recovery increases and both metrics start to tail off from ∼20% upwards. With 100% recovery of theoretical crosslinks the average precision is 0.63 and the correlation to Cα-RMSD is 0.70.

**Fig. 5.**
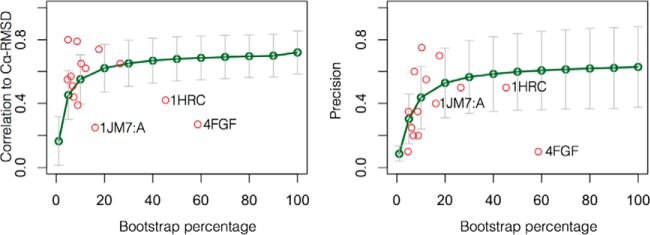
**The correlation and precision returned when using different levels of recovery tested via bootstrapping analysis.** The experimental cases from the benchmark are plotted on each graph with their experimental recovery (red circles). Major outliers: 4FGF, 1JM7:A and 1HRC are labeled. Fig. 5. (location: Results - Exploring crosslink coverage).

Plotting the precision from each experimental test case against the bootstrap analysis shows that the bootstrapped trend is largely adhered to—barring the major outlier 4FGF (Fig. 5 - labeled), for the reasons explained above (Filtering Comparative Models Using MNXL). Doing the same with the correlation shows slightly less agreement with the bootstrapping trend, however nine out of the fourteen proteins perform better than expected. Three proteins from the benchmark that perform worse than expected include 4FGF, 1JM7:A (both for reasons mentioned above) and 1HRC. The latter is the result of 11 crosslinks exceeding 33 Å in the experimentally determined structure, likely because of flexibility of this protein.

Apart from a few case specific deviations, these results suggest that our theoretical process of crosslink formation (*i.e.* the calculation of SASDs between lysine Cα-atoms that are within 33 Å from each other in the experimentally determined structure) is a good approximation. We can therefore use these results to set an experimental crosslink recovery target of ∼20%, after which return gains in performance are reduced.

#### Exploring the Maximum Bound

Within the literature there has been a range of difference maximum-bounds used for a lysine crosslinker having an 11.4 Å linker, from 25 to 35 Å (5, 11, 19, 20), with no explicit rationale for the choice. In this study we have used a maximum-bound of 33 Å. We arrived at this value by testing different cut-off values ranging from 20 to 80 Å on our benchmark. The average correlation with Cα-RMSD across the entire benchmark was found to peak across 33 - 35 Å with the maximum at ∼33 Å, however, no clear trend is observed with regards to precision (supplemental Fig. S3).

#### Discussion

Here we have shown that our MNXL score consistently performs better than NoV and SoVD in terms of correlation and precision. However, within the benchmark there is a large range of correlation (0.25 to 0.80) and precision (0.75 to 0.1), which is likely to be the result of four key factors: (1) how many lysines are on the surface of the protein; (2) how distributed the crosslinks are; (3) how flexible the protein is; and 4) how many crosslinks are recovered in the experiment. These issues could potentially inform experimental design, for example, by using additional enrichment methods when the crosslink recovery falls below 20%.

#### The Number of Lysines and the Distribution of Crosslinks

When the crosslinks are distributed across the whole protein, MNXL performs very well, exemplified by the protein 1BLF_5–333_. The percentage recovery is only 10.3%, equating to 10 crosslinks, however as they are distributed across the entire protein surface, they provide a comprehensive set of restraints to successfully model this protein. Unfortunately, it is not possible to know how evenly distributed the crosslinks are across the protein without having at least an approximate structural model. If the crosslinks are not evenly distributed, then large deviations from the experimentally-determined structure can be tolerated as in the case of 1JM7:A (Fig. 2*B*). The number of lysines a protein contains can act as a rough guide for how well the crosslinks might be distributed (supplemental Fig. S4) and therefore how well the protein might be modeled, however there are other factors to consider.

#### Considering Protein Flexibility

As crosslinking experiments are performed in solution at room temperature, the experimentally determined structure may not reflect the inherent flexibility captured in the experiment. Gathering distance restraints in this state is therefore likely to result in conflicting distance restraints, which can result in conflicting models. Within the 14 domains that were tested, the percentage of SASDs between crosslinked residues that exceed 33 Å ranges from 0 to 41.2%. For the purpose of this theoretical investigation, all SASD calculations are performed on static structures, demonstrating that considering protein flexibility when trying to derive a “native structure” is likely to play an important role in the modeling process. This disagreement between the experimentally determined structure and the crosslinking data is the main cause behind the under-performance of both 1HRC and 4FGF in the benchmark, where the “native” crystal structures perform badly.

One way of capturing protein flexibility in the modeling process, at least in part, is to use a larger maximum-bound for a crosslink restraint than the theoretical maximum. In the literature a range of cut-off distances has been used, from 25 - 35 Å, however the rational behind the choice of cut-off is not clear. A recent study by Merkley et al. (23) into crosslinked lysine distances using molecular dynamics simulations claims that it is unlikely for the backbone to shift over 8 Å for long enough for the crosslink to form, thereby setting the maximum-bound as 30 Å (22 + 8 Å). However, these studies were only performed on the nanosecond timescale and therefore excluding larger movements that might happen on the millisecond timescale. Also, this study was only performed using Euclidean distances. Our empirical approach is a different take on the problem and concludes that a value approximately around 33–35 Å is on average the optimal cut-off distance.

On the experimental side, any measures that could be taken to reduce protein dynamics during the crosslinking process would also increase the effectiveness of modeling with crosslinks. One potential avenue could be to perform crosslinking at very low temperatures.

#### Experimental Crosslink Recovery

Our bootstrapping analysis allowed us to analyze the effect of crosslink recovery on the performance of MNXL. The theoretical correlation and precision follow a similar trend and begin to tail off after ∼20% recovery. This should give the experimentalist an idea of how many crosslinks they need to collect in order to achieve the best precision.

When identifying crosslinks from the mass spectra, a false discovery rate of 5% is typically used (5, 9, 24), however a higher rate can be used in order to extract higher numbers of crosslinks. Given that the increase in modeling performance begins to tail off as the number of crosslinks increases beyond 20% recovery, we recommend against using a high false discovery rate to collect more crosslinks, as the small gains in performance are likely to be offset by the increase in potentially dubious restraints. Alternatively, the confidence score for each crosslink, calculated by programs such as xQuest (30), could provide additional weighting to the scoring procedure, which has been used in another crosslinking modeling protocol (31). However, these data are not available in the XLdb and therefore we were not able to incorporate this information in our study.

#### Monolinks and Non-accessible Crosslinks

Our benchmarking has highlighted the importance of including non-accessible crosslinks, as we find that they are responsible for the largest increase in correlation and precision. Non-accessible crosslinks capture data on the solvent accessibility of residues that is separate to the distance restraints classically used. Importantly, this opens up the possibility of using monolinks, *i.e.* crosslinks that are only linked to one lysine, allowing for more restraining information to be captured from the same number of experiments.

Our initial comparison between NoV and SoVD did not include non-accessible crosslinks because, to the best of our knowledge, this information has not been used before in the literature. The inclusion of this information brings the performance of NoV much closer to that of MNXL, however, MNXL remains more precise due to its positive scoring aspect. The same increase of performance could in theory be applied to SoVD, however, the total crosslink violating distance captured by SoVD cannot be easily combined with non-accessible crosslinks and reconciling these two data types (by finding the optimum weighting) is beyond the scope of this paper.

#### Distance Calculation

Up until now, there has been a split in the literature as to which distance has been used for modeling—SASDs or Euclidian distances. Fewer articles have used SASDs, which take longer to calculate, despite the fact that using the Euclidean distance is theoretically flawed. Furthermore, the concept of non-accessible crosslinks does not explicitly exist if Euclidean distance is used, as no account of solvent accessibility is made. It is possible to calculate solvent accessibility and then use the Euclidean distances between only solvent accessible lysines, while also considering nonaccessible crosslinks—this has the benefit of being a much faster calculation. However, given that we have shown the SASD to be a much more effective metric than the Euclidean distance, we recommend the use of SASDs when modeling using crosslinks.

#### Concluding Remarks

To conclude, our work has shown the importance of non-accessible crosslinks and the use of SASD over Euclidean distance when modeling using crosslinking restraints. Our scoring function MNXL which utilizes both of these elements among others has been shown to perform the best out of all the scoring methods tested. Additionally, our bootstrapping analysis has shown that the performance can be increased by increasing the recovery rate of crosslinks.

Future work will involve a more thorough investigation into the extent that flexibility is captured and presented by crosslinking restraints and how this might be incorporated into the modeling process, *i.e.* capturing different conformers.

## Supplementary Material

Supplemental Data

## Footnotes

^1^ The abbreviations used are:

SASD: solvent accessible surface distance
MNXL: matched and non-accessible crosslink score
NoV: number of violations
SoVD: sum of violation distances
XLdb: crosslink database
XL-MS: crosslinking coupled to mass spectrometry
BS3: bissulfosuccinimidyl suberate
DSS: disuccinimidyl suberate
SEC: size exclusion column
SCX: strong cation exchange.
