# Supplementary material for: The Importance of Non-accessible Crosslinks and Solvent Accessible Surface Distance in Modeling Proteins with Restraints From Crosslinking Mass Spectrometry
Source: Mol Cell Proteomics. 2016 May 5;15(7):2491–500. doi: 10.1074/mcp.M116.058560 (PMC4937519; doi:10.1074/mcp.M116.058560)
Supplement: Supplemental Data [file supp_15_7_2491__index.html]

The importance of non-accessible crosslinks and solvent accessible surface distance in modelling proteins with restraints from crosslinking mass spectrometry — The Importance of Non-accessible Crosslinks and Solvent Accessible Surface Distance in Modeling Proteins with Restraints From Crosslinking Mass Spectrometry — Non-accessible Crosslinks and SASD When Modelling Proteins — Supplemental Data 

# The Importance of Non-accessible Crosslinks and Solvent Accessible Surface Distance in Modeling Proteins with Restraints From Crosslinking Mass Spectrometry

## Supplemental Data

- Supplemental Figures 1-4 (.pdf, 379 KB) - Supplemental Figures 1-4 and associated figure legends
