# Supplementary material for: The Importance of Non-accessible Crosslinks and Solvent Accessible Surface Distance in Modeling Proteins with Restraints From Crosslinking Mass Spectrometry
Source: Mol Cell Proteomics. 2016 May 5;15(7):2491–500. doi: 10.1074/mcp.M116.058560 (PMC4937519; doi:10.1074/mcp.M116.058560)

Supplementary Information

Supplemental Figure Legends

Supp Figure 1. The distribution of distances between crosslinked residues from the XLdb overlaid, calculated with Xwalk (blue) and Jwalk (green).

Supp Figure 2. Example of erroneous SASD calculation by Xwalk (pink circles) compared to Jwalk (yellow path) which accounts for the main change in distributions seen in Supp Figure 1.

Supp Figure 3. The performance of MNXL when tested with a range of SASD cut-off values. The maximum correlation between the score and Cα-RMSD can be seen at 33 Å (red line).

Supp Figure 4. The correlation of the MMXL score to Cα-RMSD is itself correlated to the number of lysines present in the protein to be modelled (Pearson's correlation coefficient = 0.53)

Supplemental Figures

Supp Figure 1

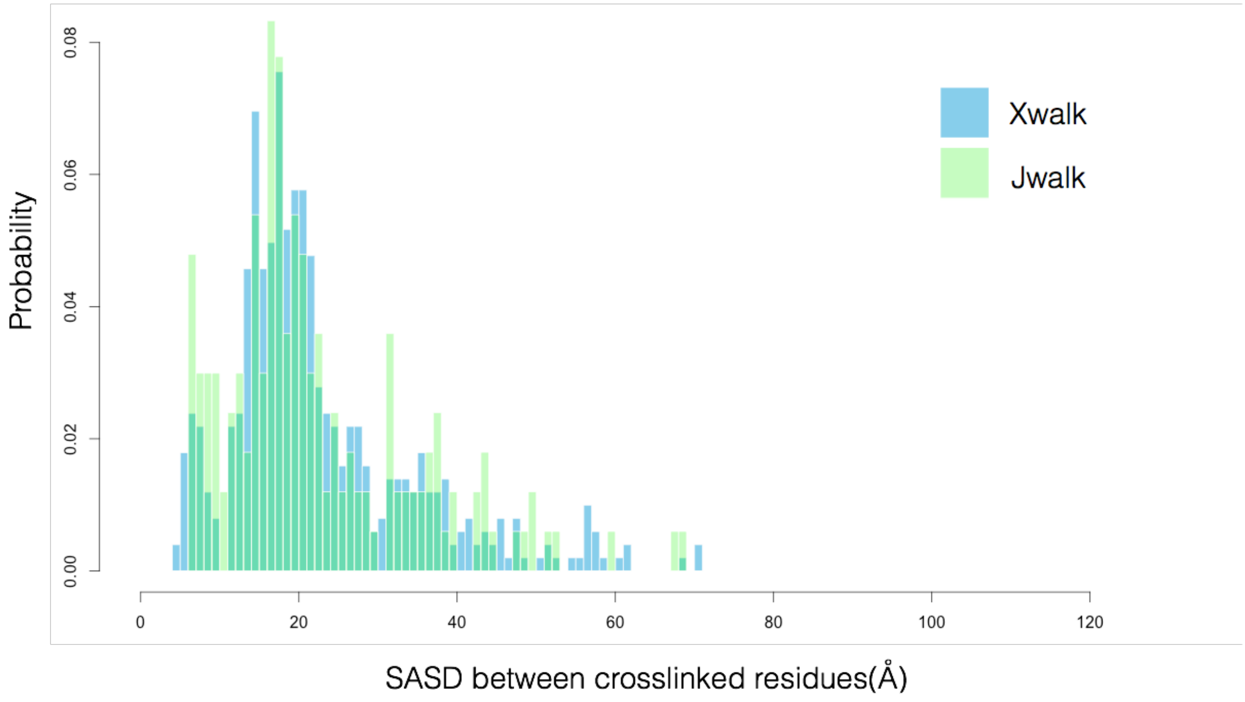

Supp Figure 2

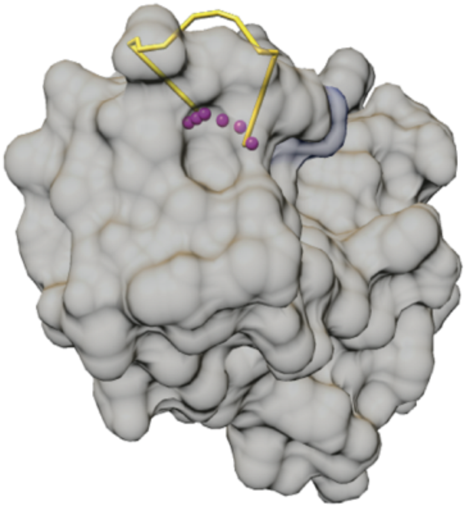

Supp figure 3

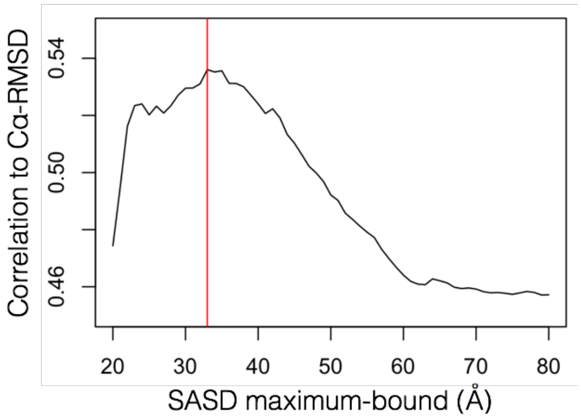

Supp Figure 4

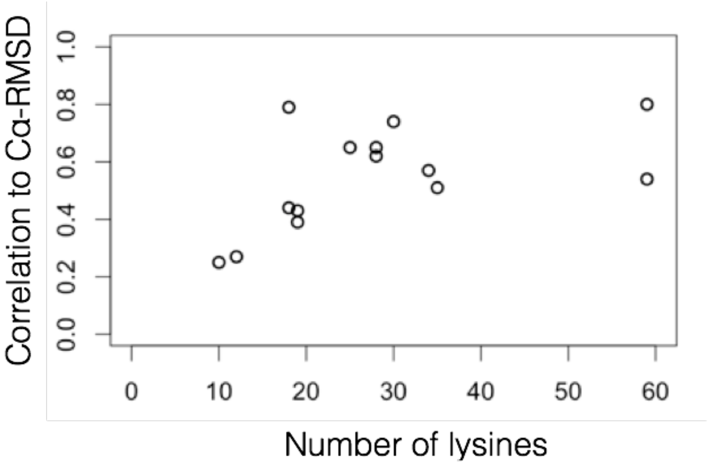

Supplement: Supplemental Data [file 10.1074_M116.058560_mcp.M116.058560-1.pdf]
